# Effects of Modified Layered Double Hydroxides on the Thermal Degradation and Combustion Behaviors of Intumescent Flame Retardant Polyethylene Nanocomposites

**DOI:** 10.3390/polym14081616

**Published:** 2022-04-15

**Authors:** Tiefeng Zhang, Chunfeng Wang, Yue Wang, Yongliang Wang, Zhidong Han

**Affiliations:** 1 School of Materials Science and Chemical Engineering, Harbin University of Science and Technology, Harbin 150040, China; zhangtiefeng79@163.com (T.Z.); chunfeng.wang@hrbust.edu.cn (C.W.); yongliangwang@hrbust.edu.cn (Y.W.); 2 School of Transportation and Vehicle Engineering, Shandong University of Technology, Zibo 255000, China; wangy@sdut.edu.cn; 3 Key Laboratory of Engineering Dielectrics and Its Application, Ministry of Education, Harbin University of Science and Technology, Harbin 150080, China

**Keywords:** layered double hydroxides, polyethylene, intumescent flame retardant, 2-carboxy ethyl (phenyl) phosphinic acid, thermal degradation, cone calorimeter

## Abstract

The flame retardancy of layered double hydroxides (LDHs) correlates with their structure and dispersion in a polymeric matrix. To improve the flame retardant effectiveness of Mg-Al LDH in polyethylene (PE), 2-carboxy ethyl (phenyl) phosphinic acid (CEPPA) was adopted as a flame retardant modifier to prepare CEPPA-intercalated LDH (CLDH) by the regeneration method, which was then exfoliated in PE by melt blending in the form of a masterbatch prepared from solution mixing. By compounding CLDH with intumescent flame retardant (IFR) composed of ammonium polyphosphate (APP) and pentaerythritol (PER), the thermal degradation and combustion behaviors of the flame retardant PE-based composites were investigated to reveal the flame retardant mechanism between CLDH and IFR in PE. The reactions between CLDH and IFR were revealed to make a predominant contribution to the compact and fully developed char of PE/IFR/CLDH, which enhanced the flame retardancy of the composites.

## 1. Introduction

The fire safety of polyethylene (PE) in many fields such as wire and cables has attracted great concern due to its high flammability and the large heat release during its combustion. Recently, Ghomi et al. [1] delivered a review on the flame retardancy of PE composites and described the progress of some well-known flame retardant families in PE, including phosphorous, melamine, nitrogen, inorganic hydroxides, boron, and silicon. Considering the potential threat of some flame retardants on the environment and human health, halogen-free flame retardants with non-toxic and environmentally friendly characteristics will be a step towards future research and development [2].

Layered double hydroxides (LDHs), a kind of anionic clay, are composed of positively charged brucite-like layers and charge-compensating anions within interlayer regions [3]. The application of LDHs has extended into various fields, among which the increasing interest comes from the flame retardant materials [4,5,6]. As a promising type of environmentally friendly flame retardant additive, the flame retardancy of LDHs is related to their element composition [7], interlayer ions [8], and particle size [9]. The layered structure and anion exchange capacity of LDHs make it possible to tailor the flame retardancy by the intercalation of different compounds, such as P_3_O_10_^5−^ [10], phosphorylated cellulose [11], ammonium polyphosphate (APP) [12], polyborosiloxane [13], and sodium lignosulfonate [14]. Furthermore, synergistic effects have also been reported between LDHs and APP [15], montmorillonite (MMT) [16], expandable graphite (EG) [17], intumescent flame retardant (IFR) [18], and zinc borate (BZn) [19].

The modification and dispersion of LDHs were found to play important roles in the flame retardancy of the composites [3,13]. Interlayer modification can alleviate the van der Waals forces and electrostatic forces between the interlayers and improve the dispersion of LDH in polymers. In some cases, exfoliated nanocomposites were obtained due to the enlarged interlayer spacing by interlayer modification. It should be noted that the preparation strategy also significantly influences the dispersion of LDHs [3]. Melt blending and solution mixing are commonly applied to prepare polymer/LDHs composites [20], on the basis of which some new methods have been developed, such as the aqueous miscible organic solvent treatment (AMOST) by Qiu et al. [21]. 

From the perspective of industrial manufacturing, melt blending is preferred to prepare PE/LDHs composites. Taking this into account, interlayer modification and compounding with a synergistic flame retardant would be expected to simultaneously enhance the dispersion of LDHs and the flame retardancy of PE/LDHs composites. Gao et al. [22] used LDHs with different interlayer anions in high-density polyethylene (HDPE) and disclosed the influence of anions on the flame retardancy of the nanocomposites. The peak heat release rate (pHRR) of the nanocomposites with 40 wt.% LDH was reduced by 24%, 41%, 48%, and 54% for HDPE/Zn_2_Al–Cl, HDPE/Zn_2_Al–CO_3_, HDPE/Zn_2_Al–NO_3_, and HDPE/Zn_2_Al–SO_4_, respectively. Costa et al. [23] exfoliated Mg-Al LDH intercalated with sodium dodecylbenzenesulfonate (SDBS) in low density polyethylene (LDPE) by the melt-compounding method and reported the value of limiting the oxygen index (LOI) to about 22.0 and decreased the heat release of the nanocomposites with 16.20 wt.% LDH. Ye et al. [24] investigated the effects of stearate anion (SA) and dodecyl sulfate anion (DS)-intercalated LDH on the flame retardant properties of LDPE and reported a better flame retardance and thermal stability of the nanocomposites with SA-intercalated LDH. Khanal et al. [25] revealed the synergistic effect of LDHs with IFR in HDPE and reported that the pHRR decreased by 12% for the composites with 3 wt.% LDH and 22 wt.% IFR in comparison to the composites with 25% IFR.

So far, an environmentally friendly halogen-free flame retardant with high effectiveness is still one of the great challenges for PE, while the processing of LDHs and their applications in PE needs to be further studied in relation to the flame retardancy and modification. In this work, 2-carboxy ethyl (phenyl) phosphinic acid (CEPPA) was adopted as a flame retardant modifier to prepare CEPPA-intercalated Mg-Al LDH (CLDH) by the regeneration method. CLDH was exfoliated in maleated polyethylene (PEgMA) to obtain a CLDH/PEgMA masterbatch by solution mixing. The masterbatch was then compounded with IFR (using APP and PER as components) and applied in PE to obtaine flame retardant composites by melt blending. The thermal degradation and combustion behaviors of the flame retardant composites were investigated to reveal the synergistic mechanism between CLDH and IFR in PE. The strategy proposed in this work would be helpful for the development of halogen-free flame retardant polyethylene with a high flame retardant efficiency.

## 2. Materials and Methods

### 2.1. Materials

Linear low-density polyethylene (LLDPE, 7042) was a commercial product of the Daqing petrochemical company of Petro China (Daqing, China). Maleated polyethylene (PEgMA) was purchased from Nengzhiguang new materials company (Ningbo, China). Mg-Al LDH (Mg_6_Al_2_(OH)_16_CO_3_·2H_2_O) was obtained from Huashi Huadong plastics additive company (Jiangyin, China). 2-carboxy ethyl (phenyl) phosphinic acid (CEPPA) was obtained from Shangdong Xingqiang flame retardant technology Co., Ltd. (Dezhou, China). Sodium dodecyl sulfate (SDS) was obtained from Tianjin Guangfu research institution of fine chemical engineering (Tianjin, China). Ammonium polyphosphate (APP, *n* > 1000) was obtained from Hangzhou JLS Flame Retardants Chemical Co., Ltd. (Hangzhou, China). Pentaerythritol (PER) was obtained from Tianjin Nankai Chemical Factory (Tianjin, China). All materials were used as received without any further treatment. 

### 2.2. Preparation Methods

Synthesis of CEPPA-intercalated LDH (CLDH). The regeneration method was adopted to synthesize CLDH as shown in Figure S1. In general, LDH was calcined in a muffle oven at 500 °C for 6 h to obtain layered double oxide (LDO). A total of 2 g LDO was dispersed in 50 mL decarbonated water and stirred for 30 min under a nitrogen atmosphere. A total of 1 g CEPPA was added into the solution and the pH of the solution was adjusted to about 10 using NaOH solution. The solution was then refluxed for 4 h and the product was obtained after centrifuging, filtering, and drying. SDS-intercalated LDH (SLDH) was also synthesized by the same method. Three kinds of LDHs, including LDH, SLDH, and CLDH were used in order to compare the influences of intercalated compounds on the flame retardancy of the composites.

Preparation of CLDH/PEgMA masterbatch. The masterbatch was prepared by the solution mixing method. As an example, 5 g CLDH was ultrasonically treated for 1 h in the solvent of xylene. Then, 10 g PEgMA was added into the solution and refluxed for 3 h. The hot solution was quickly poured into the mixture of water and ethanol (volume ratio of 1:1). The precipitate was collected and dried to obtain the CLDH/PEgMA masterbatch. 

Preparation of flame retardant composites. The composites were prepared by melt blending according to Table S1 with the rheometer (Harbin Hapu Electric Technology Ltd., Harbin, China). The PE and CLDH/PEgMA masterbatch were added into the rheometer at 423 K and blended for 2 min at a speed of 40 rpm/min and 4 min at a speed of 80 rpm/min. Then, IFR with components of APP and PER (mass ratio of 2:1) was added to the rheometer and blended according to the same procedure. The composites were obtained after hot-pressing under 10 MPa.

### 2.3. Characterization

X-ray diffraction (XRD) was performed on an X-ray diffractometer (X’Pert PRO, PANalytical, Almelo, The Netherlands) at 40 kV and 80 mA with Cu Kα (λ = 0.154 nm) at the scanning rate of 2°/min in the range of 2°–40°. The morphology of the composites was observed by scanning electron microscopy (SEM, Sirion200, FEI, Hillsboro, OR, USA) and transmission electron microscopy (TEM, JEM-2100, JEOL, Tokushima, Japan). The sample for SEM was cryo-fractured in liquid nitrogen and the fractured surface was metallized. The FTIR spectra of the composites were recorded by using attenuated total reflection flourier transformed infrared spectroscopy (ATR-FTIR, Spectrum 400, Perkin Elmer, Waltham, MA, USA). The combustion behaviors were evaluated according to ISO 5660 on a CONE calorimetry (FTT, West Sussex, UK) at a heat flux of 35 kW/m^2^ with specimens of 50 mm × 50 mm × 3 mm. The surface elemental compositions of the char residues were analyzed by X-ray photoelectron spectroscopy (XPS) on a PHI Quantera-II SXM (Ulvac-PHI, Chigasaki, Japan). 

Thermogravimetric analysis (TGA) was performed on Q500 (TA, New Castle, DE, USA) at a heating rate of 10 °C/min from 50 °C to 700 °C in a nitrogen atmosphere. The theoretical TG curve was calculated from the TG curves of individual components by their contents according to Equation (1). The weight difference (ΔWeight) was calculated to investigate the compounding effects on the weight loss by subtracting the theoretical TG curves from the experimental ones according to Equation (2).

TG_theo,AB_ = *w*_A_ × TG_exp,A_ + *w*_B_ × TG_exp,B_ (1)

where *w*_A_ and *w*_B_ are the mass contents of components A and B in AB composites, respectively; and TG_exp,A_ and TG_exp,B_ represent the weight versus temperature curves of the component A and B by TGA method, respectively.

ΔWeight = TG_exp,AB_ − TG_theo,AB_ (2)

where TG_exp,AB_ represents the weight versus temperature curve of AB composites by the TGA method.

## 3. Results

### 3.1. Structure of Modified LDH and Its Nanocomposites

**Figure 1:**
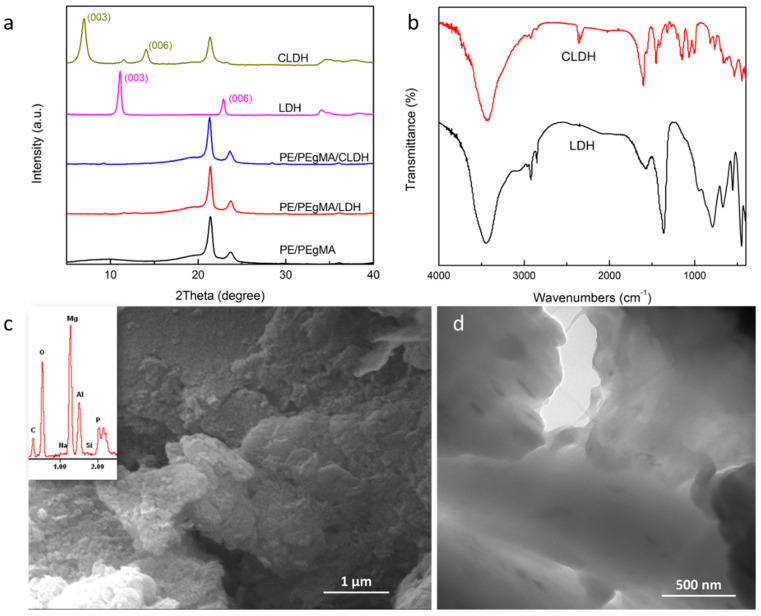
(**a**) XRD patterns of LDH, CLDH, and their composites; (**b**) FTIR spectra of LDH and CLDH; (**c**) SEM micrograph of CLDH inserted with EDS spectrum of CLDH; (**d**) TEM micrograph of PE/PEgMA/CLDH.

CEPPA is an efficient phosphorus-containing flame retardant and can be used as a reactive component in IFR [26,27,28]. To improve the flame retardancy of LDH and its dispersion in PE, CLDH was prepared with an industrial LDH product by the regeneration method. Figure 1a compares the XRD patterns of LDH and CLDH. The (003) diffraction reflection of LDH at 2θ = 11.1° revealed the d-value of 0.79 nm, which was consistent with that of carbonate-intercalated Mg-AL LDH. The (003) reflection peak of CLDH shifted to 2θ = 7.0° with a d-value of 1.26 nm, which illustrated the enlarged interlayer space of CLDH and the successful intercalation of CEPPA into the interlayer space. The regeneration method was adopted in this work because of the inertia of the carbonate of industrial LDH in the ion exchanging reaction. The interlayer space of CLDH by the regeneration method was consistent with that of CEPPA-intercalated Ni-Al LDH by the ion-exchanging of nitrate in LDH [29]. By using the regeneration method, SLDH was obtained and showed a d-value of 2.60 nm. 

Figure 1b shows the FTIR spectra of LDH and CLDH. The spectrum of LDH presents the absorption bands of O–H stretching at 3452 cm^−1^, the band of hydroxyl deformation at 1584 cm^−1^, the band of CO_3_^2−^ vibration at 1366 cm^−1^, and the bands of Mg–O and Al–O vibrations in the range of 800–400 cm^−1^ [3]. The bands at 2920 cm^−1^, 2850 cm^−1^, and 940 cm^−1^ indicate that there were some organic species in the industrial product of LDH. The spectrum of CLDH presents the absorption bands of CEPPA at 1604 cm^−1^, 1457 cm^−1^, and 1154 cm^−1^. The characteristic bands at 1214 cm^−1^, 1069 cm^−1^, and 997 cm^−1^ were assigned to P–C, P–O, and P–OH vibrations [27,29]. The disappearance of CO_3_^2−^ vibration at 1366 cm^−1^ revealed the ion substitution by CEPPA. The shifts of O–H stretching vibration to 3426 cm^−1^ and hydroxyl deformation to 1563 cm^−1^ revealed the enhanced hydrogen bonding between the interlayer water and the hydroxyl groups of the host layers by CEPPA, as proved by the vibration at 2363 cm^−1^. Accordingly, CEPPA was successfully intercalated into the interlayer galleries of LDH, which induced the ion bonding and hydrogen bonding between CEPPA and the host layers. 

The morphology of CLDH is shown in Figure 1c. The typically layered structure was observed with some exfoliated layers on the surface. As characterized by EDS in Table S2, the molar ratio of Mg^2+^ and Al^3+^ in CLDH was about 2.09 and the molar percentage of P was about 2.83 at.%. Figure 1d shows the TEM micrograph of PE/PEgMA/CLDH composites with 3 wt.% CLDH and illustrates a good dispersion of CLDH in PE/PEgMA with exfoliated layers. As widely accepted, the disappearance of characteristic diffraction peaks of layered inorganic compounds indicates the formation of the exfoliated nanocomposites [30]. Thus, it can be seen in Figure 1a that the disappeared diffraction peaks of LDH and CLDH in the patterns of PE/PEgMA/LDH and PE/PEgMA/CLDH reveal the formation of the nanocomposites with the exfoliated structure. This result confirms the positive role of the preparation strategy in delaminating the layers of LDHs. By using the same method, we obtained the intercalated LDH with sodium dodecyl sulfate (SLDH) and delaminated it in PE [31]. In the following sections, the effects of LDH, SLDH, and CLDH on the thermal degradation and combustion behaviors of the flame retardant PE are investigated to reveal the synergistic mechanism between CLDH and IFR. 

### 3.2. Thermal Degradation of LDHs, IFR/LDHs, and PE/IFR/LDHs

**Figure 2:**
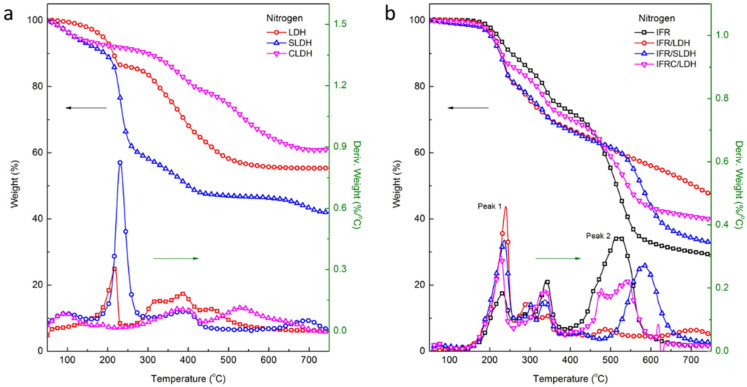
TG curves of (**a**) LDHs and (**b**) IFR/LDHs.

The thermal decomposition of LDH is characterized by the release of interlayer water, the decomposition of intercalated anions, and the dehydroxylate of the laminate structure. As shown in Figure 2a, the weight loss of LDH, SLDH, and CLDH at 260 °C was 14.0%, 38.1%, and 8.5%, respectively. SLDH contained more interlayer water while CLDH contained less water. LDH, SLDH, and CLDH underwent 29.5%, 15.2%, and 22.4% weight loss in the temperature range of 260 °C and 550 °C, and left residues of 55.6%, 43.5%, and 61.0% at 700 °C, respectively. 

When compounded with IFR, LDHs exerted different influences on the thermal degradation of IFR/LDHs. As shown in Figure 2b, the TG curves of IFR/LDHs presented three thermal degradation regions. The first region in the temperature range of 150 °C and 260 °C was related to the decomposition of APP and the interlayer water release of LDHs. LDHs gave rise to a higher weight loss and loss rate in this region, among which LDH contributed more and CLDH less. The second region occurred in the temperature range of 260 °C and 400 °C, where APP reacted with PER and the intumescent char was formed. The interaction between LDHs and IFR made the process complicated. IFR/LDH and IFR/SLDH showed two degradation peaks in the region while IFR/CLDH showed one peak and a higher loss rate. The thermal degradation of intumescent char happened in the third region of 400 °C and 700 °C. IFR/LDHs delayed the char degradation and left more residues.

According to the TG data in Table S3, IFR/LDHs showed a lower temperature at 5% weight loss (T_5_) and a higher loss rate of the first peak (R_p1_), which revealed their lower thermal stability compared to IFR. Meanwhile, the higher temperature corresponding to the second maximal peak (T_p2_) and the lower loss rate (R_p2_) indicated the better thermal stability of the intumescent char of IFR/LDHs than that of IFR. The ΔWeight curves in Figure S2 show the greater weight loss of IFR/LDHs in the temperature range of 150 °C and 460 °C compared to the theoretical prediction, and more residue reserved in the temperature range of 460 °C and 700 °C. It follows that the interactions between LDHs and IFR promoted the formation of intumescent char and improved the thermal stability of the char at high temperature.

**Figure 3:**
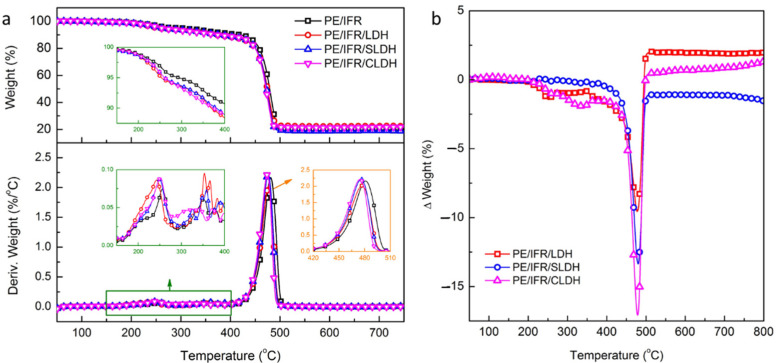
(**a**) TG curves and (**b**) ΔWeight versus temperature curves of PE/IFR/LDHs.

IFR and LDHs were added into the PE and the TG curves of PE/IFR/LDHs shown in Figure 3. As can be seen in Figure 3a, the thermal stability of PE/IFR/LDHs was lower than that of PE/IFR with a lower T_5_, which was 30 °C lower for PE/IFR/LDH and 39 °C lower for PE/IFR/SLDH and PE/IFR/CLDH. Three regions were observed during the thermal degradation process of PE/IFR/LDHs, which was very similar to IFR/LDHs. In comparison with PE/IFR, PE/IFR/LDHs presented a lower peak temperature and more weight loss in the first and the second region, which was related to the intumescent process of IFR/LDHs. The intumescent char formed around 400 °C and about 10% weight loss occurred. Concerning the temperature at 10% weight loss (T_10_), the thermal stability of PE/IFR/LDHs was about 45 °C lower than that of PE/IFR. About 70% weight loss happened in the third region, which was mainly attributed to the degradation of PE. PE/IFR/LDHs showed a lower peak temperature than PE/IFR while both PE/IFR/LDH and PE/IFR/CLDH showed more residues at 550 °C. The ΔWeight versus temperature curves in Figure 3b showed more weight loss of PE/IFR/SLDH in the whole temperature range while PE/IFR/LDH and PE/IFR/CLDH showed more residues reserved in the temperature range of 500 °C and 800 °C. It follows that three kinds of LDHs exerted different influences on the thermal degradation of PE/IFR/LDHs. LDH and CLDH were revealed to enhance the charring of the composites because the ΔWeight values of PE/IFR/LDH and PE/IFR/CLDH at 750 °C were positive, which were 1.94% and 1.03%, respectively. Comparatively, SLDH had negative effects on charring according to the ΔWeight value of −1.27% at 750 °C. Due to the different charring effects of LDHs, PE/IFR/LDHs would show different flame retardancy and combustion behaviors.

### 3.3. Combustion Behaviors of PE/IFR/LDHs

**Figure 4:**
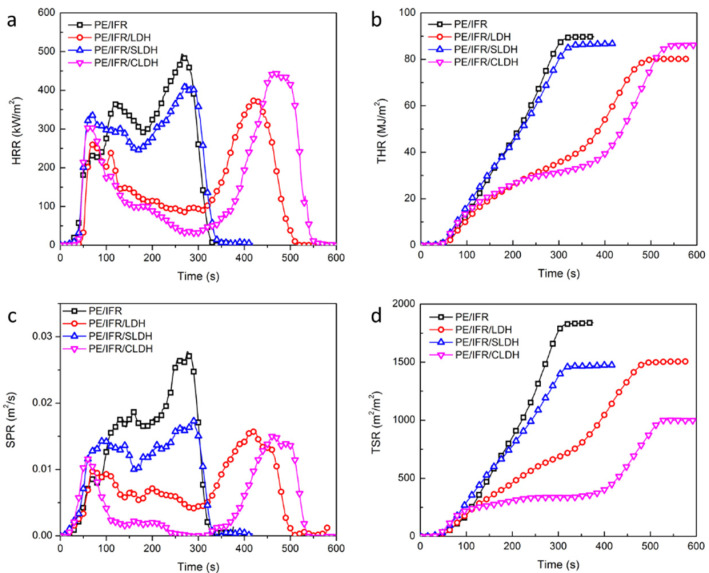
The results of CONE, (**a**) HRR curves, (**b**) THR curves, (**c**) SPR curves, and (**d**) TSR curves.

The flame retardancy and smoke suppression properties of LDHs have been reported to be superior to aluminum hydroxide and magnesium hydroxide [32], and the synergistic effect between LDHs with IFR was also reported in HDPE [25]. In order to investigate the effects of the intercalation compound on the combustion behaviors of flame retardant composites, CONE testing was performed on PE/IFR/LDHs composites with three kinds of LDHs. The CONE results are shown in Figure 4 and Table S4. The HRR curves of the flame retardant composites presented double-peak characteristics during their combustion. The first peak can be attributed to the formation of intumescent char while the second one can be attributed to the degradation of the intumescent char and PE. The gap between the two peaks, to some extent, reflects the effectiveness of the intumescent in preventing further combustion of the composites. As can be seen, the intumescent char of PE/IFR/CLDH exerted the most effective action of flame retardancy. Viewing the HRR curves, PE/IFR/LDH showed the lowest pHRR value, which was reduced by 25% in comparison with PE/IFR, while PE/IFR/CLDH showed the longest time to peak HRR, which was about 1.8 times longer than PE/IFR. PE/IFR/LDH and PE/IFR/CLDH also showed a lower total heat release (THR) and smoke production rate (SPR). It should be noted that PE/IFR/CLDH presented the lowest average value of HRR. Furthermore, PE/IFR/CLDH also presented the lowest value of total smoke release (TSR).

**Figure 5:**
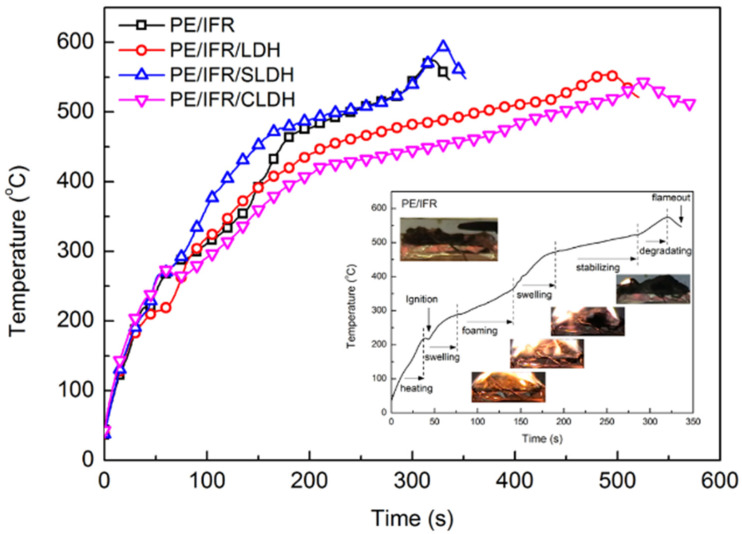
Temperature versus time curves of the flame retardant composites during CONE testing.

The thermal shielding performance of the intumescent char was investigated by in-line temperature measurements on a cone calorimeter [33,34]. The temperature versus time curves of the flame retardant composites during CONE testing are shown in Figure 5. Several stages were observed during the combustion of PE/IFR, including heating, foaming, swelling, stabilizing, and degradation. PE/IFR/LDHs showed similar temperature profiles with these stages. The evolution of the temperature profile can be well interpreted by the intumescent process taking place during testing. A fully developed intumescent char formed at about 178 s for PE/IFR, 202 s for PE/IFR/LDH, 170 s for PE/IFR/SLDH, and 217 s for PE/IFR/CLDH. The temperature corresponding to the char formation was 464 °C for PE/IFR, 439 °C for PE/IFR/LDH, 474 °C for PE/IFR/SLDH, and 423 °C for PE/IFR/CLDH. Since the thermocouples were embedded in the intumescent char, the lower temperature suggests better thermal shielding effects of the intumescent char. Furthermore, the time that was consumed to achieve 500 °C (t_500C_) reflects the thermal stability of the intumescent char. The following order of t_500C_ was revealed, PE/IFR/CLDH > PE/IFR/LDH > PE/IFR > PE/IFR/SLDH. Consequently, the intumescent char of PE/IFR/CLDH presented the best thermal shielding effectiveness and thermal stability among the four kinds of flame retardant composites.

### 3.4. Char Structure after CONE Testing

**Figure 6:**
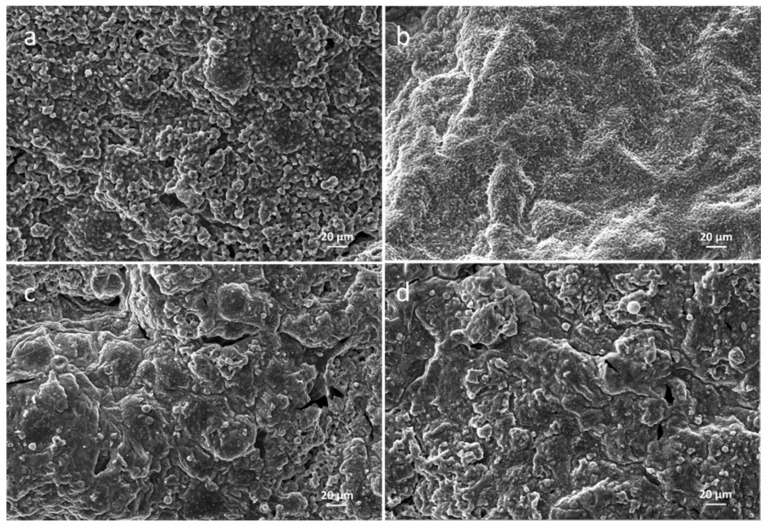
SEM micrographs of the char after CONE Testing of the composites. (**a**) PE/IFR, (**b**) PE/IFR/LDH, (**c**) PE/IFR/SLDH, and (**d**) PE/IFR/CLDH.

The intumescent char functions as a barrier to heat and mass transfer during the combustion, the structure of which is of great importance in the flame retardancy of the composites. The morphological structure of the char after CONE testing was photographed by a digital camera in Figure S3 and observed by SEM in Figure 6, respectively. The char of PE/IFR showed the rough surface of a stacked structure with cracks and holes. In comparison with PE/IFR, the char of PE/IFR/SLDH seemed underdeveloped and sunk to some extent. Remarkable cracks and holes were observed on the char surface, which would impair the effectiveness of the char. In contrast, the well-developed char of PE/IFR/LDH and of PE/IFR/CLDH was found with better charring and intumescent phenomena. The char of PE/IFR/LDH showed a smooth surface without obvious cracks and holes while that of PE/IFR/CLDH showed a relatively smooth surface with small cracks and holes. 

**Figure 7:**
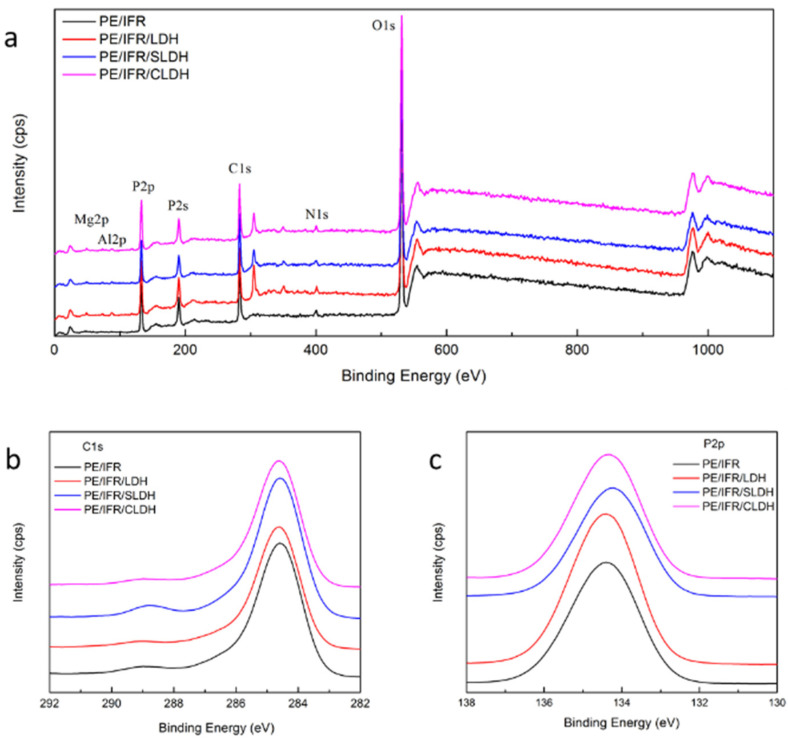
XPS spectra of the char after CONE testing of the flame retardant composites. (**a**) Survey spectra, (**b**) C1s spectra, and (**c**) P2p spectra.

Accordingly, the compact and fully developed char of PE/IFR/LDH and PE/IFR/CLDH can exert the barrier function more effectively, which was reflected in the significantly reduced HRR and SPR during their combustion. The char structure was further investigated by XPS, as shown in Figure 7. By comparing the survey spectra of the composites in Figure 7a, elements of Mg and Al presented in the spectra of PE/IFR/LDHs due to the addition of LDHs. Elements of C, O, N, and P were found in all the composites due to the compositions of PE and IFR. The charring of the composites after combustion was obviously thanks to the relatively high content of the C element in Table S5, which followed the order of PE/IFR/SLDH > PE/IFR > PE/IFR/CLDH > PE/IFR/LDH. Owing to the higher content of P and Mg, the lower C content was found in the char of PE/IFR/LDH and PE/IFR/CLDH, which suggested the accumulation of LDH and APP on the surface during the combustion. The ratio of Mg:C and P:C provided the evidence, as shown in Table S5. The char of the composites showed similar C1s spectra in Figure 7b except that a more remarkable peak at 288.7 eV was found in the spectrum of PE/IFR/SLDH. Such a phenomenon could be explained by the oxidation of the char due to its lower thermal stability. While observing P2p spectra in Figure 7c, the peak position of PE/IFR, PE/IFR/LDH, PE/IFR/SLDH, and PE/IFR/CLDH was located at 134.4 eV, 134.4 eV, 134.2 eV, and 134.3 eV, respectively. The results revealed the structure of P–O–C in the char. Considering the broadened peak of P2p as well as the lower binding energy for PE/IFR/SLDH and PE/IFR/CLDH, the structure of P = O was proposed in the char, which was mainly formed due to the degradation and reaction of APP. 

### 3.5. Flame Retardant Mechanism

**Figure 8:**
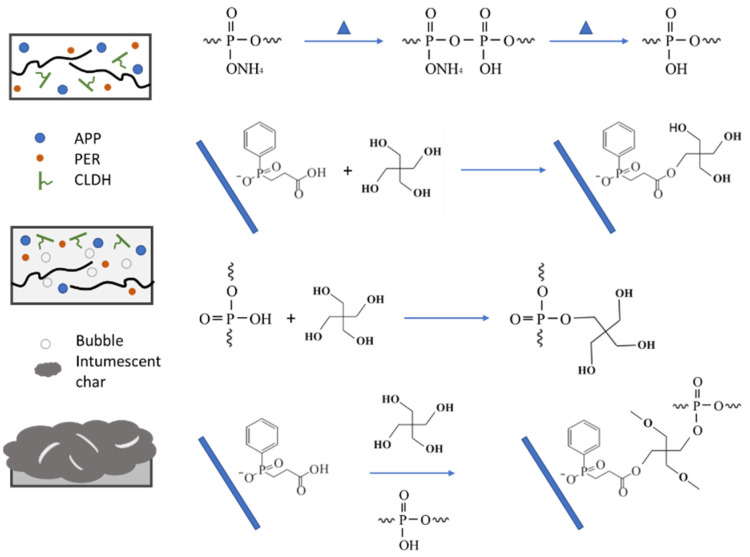
Schematic diagram of the flame retardant mechanism of PE/IFR/CLDH.

Previously, we discussed the thermal degradation and combustion behaviors of the flame retardant PE with IFR and LDHs. By comparative investigations with three kinds of LDHs, the intercalated compounds were found to exert strong influences on the flame retardant effectiveness of LDHs. With CEPPA as an intercalated compound, we obtained the significant enhancement of the flame retardancy of PE in combination with IFR and CLDH. Comparing LDH and SLDH, CLDH showed superiority in reducing HRR and SPR during combustion. These two parameters are of predominant importance in evaluating the fire safety of the composites. Accordingly, we focused on discussing the flame retardant mechanism of PE/IFR/CLDH in the section, as proposed in Figure 8. 

The flame retardant mechanism of PE/IFR/CLDH can be related to the decomposition of CLDH, the reaction of IFR, and the interaction between CLDH and IFR. During the degradation and combustion process of PE/IFR, APP decomposed to form polyphosphoric acid and ammonia, and then the polyphosphoric acid reacted with PER to form a phosphate ester [35,36]. The phosphate ester underwent dehydration to form intumescent char, which functioned as a barrier to endow flame retardancy to PE. The decomposition of CLDH was processed by the release of interlayer water, the decomposition of intercalated anions, and the dehydroxylate of laminate structure [3,23,37]. During these processes, the heat absorbed, and the inert gas released became the key factors for CLDH to exert its flame retardant functions, as well as the resulted metal oxide and char. Meanwhile, the carboxyl and phosphonic acid groups in CEPPA reacted with the hydroxyl groups of PER, which were in favor of the enhancement of intumescent char. 

Accordingly, the function of CLHD that enhanced the flame retardancy of PE/IFR can be explained in the condensed phase and in the gas phase. In the condensed phase, CLDH reacted with IFR and promoted the formation of intumescent char as well as the charring of the nanocomposites. The char acted as a barrier to heat and mass transfer and restrained HRR and SPR. In the gas phase, the nonflammable gases derived from IFR and CLDH, such as NH_3_ and H_2_O, diluted the concentration of oxygen and flammable gases from the thermal degradation of PE, which inhibited the combustion and suppressed the evolved heat. 

## 4. Conclusions

CEPPA as an efficient phosphorus-containing flame retardant was intercalated into the interlayer galleries of Mg-Al LDH to prepare CLDH by the regeneration method. CLDH showed interlayer spacing of 1.26 nm and was successfully exfoliated in PE according to the proposed preparation strategy. Three kinds of LDHs, including LDH, SLDH, and CLDH were compounded with IFR in flame retardant PE to reveal their influences on the thermal degradation and combustion behaviors of the composites. According to the TG results, the interactions between LDHs and IFR promoted the formation of intumescent char and improved the thermal stability of the char at a high temperature. LDH and CLDH enhanced the charring of the composites while SLDH had negative effects on charring. The CONE results testified the most effective action of the intumescent char of PE/IFR/CLDH among the four flame retardant composites by showing the lowest average value of HRR and the lowest TSR value. Meanwhile, the in-line temperature results provided further evidence for the enhanced thermal shielding effectiveness and thermal stability of the intumescent char of PE/IFR/CLDH. The reactions between CLDH and IFR were revealed to make a predominant contribution to the compact and fully developed char of PE/IFR/CLDH, which enhanced the flame retardancy of the composites.

## Supplementary Materials

The following supporting information can be downloaded at: https://www.mdpi.com/article/10.3390/polym14081616/s1, Figure S1: schematic route to synthesis of CLDH by regeneration method; Figure S2: ΔWeight versus temperature curves of IFR/LDHs; Figure S3: The digital photographs of the char after CONE Testing of the composites. (a) PE/IFR, (b) PE/IFR/LDH, (c) PE/IFR/SLDH and (d) PE/IFR/CLDH; Table S1: formulations of the flame retardant composites; Table S2: EDS data of CLDH; Table S3: TG data of LDHs and IFR/LDHs; Table S4: data of CONE; Table S5: data of XPS.
