# Supplementary material for: Effects of Modified Layered Double Hydroxides on the Thermal Degradation and Combustion Behaviors of Intumescent Flame Retardant Polyethylene Nanocomposites
Source: Polymers (Basel). 2022 Apr 15;14(8):1616. doi: 10.3390/polym14081616 (PMC9030746; doi:10.3390/polym14081616)
Supplement: Supplementary file 1 [file polymers-14-01616-s001.zip › polymers-1658518-supplementary.pdf]

polymers

MDPI

*Supplementary data*

# Effects of Modified Layered Double Hydroxides on the Thermal Degradation and Combustion Behaviors of Intumescent Flame Retardant Polyethylene Nanocomposites

**Tiefeng Zhang [1], Chunfeng Wang [1], Yue Wang [2], Yongliang Wang [1] and Zhidong Han [1,3,*]**

[1] School of Materials Science and Chemical Engineering, Harbin University of Science and Technology, Harbin 150040, China; zhangtiefeng79@163.com (T.Z.); chunfeng.wang@hrbust.edu.cn (C.W.); yongliangwang@hrbust.edu.cn (Y.W.)
[2] School of Transportation and Vehicle Engineering, Shandong University of Technology, Zibo 255000, China; wangy@sdut.edu.cn
[3] Key Laboratory of Engineering Dielectrics and Its Application, Ministry of Education, Harbin University of Science and Technology, Harbin 150080, China
* Correspondence: zhidong.han@hrbust.edu.cn

2-carboxy ethyl (phenyl) phosphinic acid (CEPPA) is an efficient phosphorus-containing flame retardant and a reactive component in intumescent flame retardant (IFR). To improve the flame retardant effectiveness of LDH and the reactivity with IFR, CEPPA intercalated LDH (CLDH) was synthesized by regeneration method as shown in Figure S1.

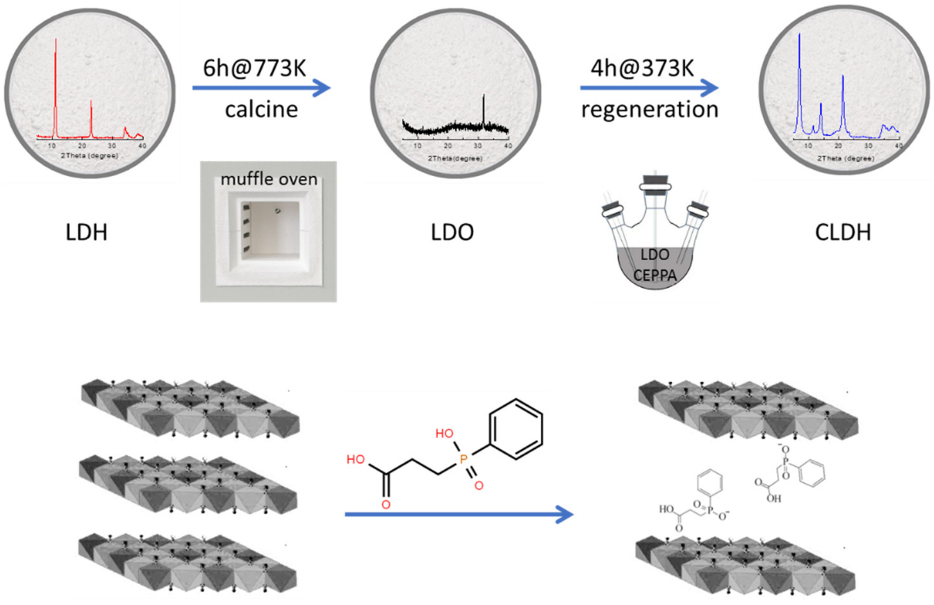


**Figure S1.** Schematic route to synthesis of CLDH by regeneration method.

CLDH was then compounded with intumescent flame retardant (IFR) to enhance the flame retardancy of polyethylene (PE). The composites were prepared by melt blending according to Table 1. Three kinds of LDHs, including LDH, SDS intercalated LDH (SLDH) and CLDH were used in order to compare the influences of intercalated compounds on the flame retardancy of the composites.

**Table S1.** Formulations of the flame retardant composites.

| Sample | PE (wt.%) | PEgMA (wt.%) | APP (wt.%) | PER (wt.%) | LDHs (wt.%) |
|---|---|---|---|---|---|
| PE/IFR | 60 | 10 | 20 | 10 | -- |
| PE/IFR/LDH | 57 | 10 | 20 | 10 | 3 (LDH) |
| PE/IFR/SLDH | 57 | 10 | 20 | 10 | 3 (SLDH) |
| PE/IFR/CLDH | 57 | 10 | 20 | 10 | 3 (CLDH) |

As characterized by EDS, the molar percentage of elements in CLDH was shown in Table S2.

**Table S2.** EDS data of CLDH.

| Element | Molar percentage (at.%) |
|---|---|
| C | 36.72 |
| O | 40.32 |
| Mg | 13.38 |
| Al | 6.40 |
| P | 2.83 |
| Na | 0.35 |

Thermogravimetric analysis (TGA) was performed at a heating rate of 10 °C/min from 50°C to 700°C in nitrogen atmosphere. TG data of LDHs and IFR/LDHs were shown in Table S3.

**Table S3.** TG data of LDHs and IFR/LDHs.

| Sample | $T_5$ (°C) | $T_{10}$ (°C) | Peak 1 | | Peak 2 | | Residue at 700 °C (%) |
|---|---|---|---|---|---|---|---|
| | | | $T_{p1}$ (°C) | $R_{p1}$ (%/°C) | $T_{p2}$ (°C) | $R_{p2}$ (%/°C) | |
| LDH | 189 | 214 | 217 | 0.31 | 386 | 0.19 | 55.6 |
| SLDH | 120 | 193 | 232 | 0.82 | 383 | 0.10 | 43.5 |
| CLDH | 122 | 304 | 378 | 0.11 | 535 | 0.11 | 61.0 |
| IFR | 222 | 254 | 232 | 0.19 | 521 | 0.36 | 30.0 |
| IFR/LDH | 211 | 230 | 240 | 0.46 | - | - | 50.6 |
| IFR/SLDH | 204 | 225 | 236 | 0.35 | 581 | 0.27 | 34.6 |
| IFR/CLDH | 209 | 229 | 228 | 0.31 | 538 | 0.23 | 40.8 |

Figure S2. illustrated the ΔWeight curves to further investigate the effects of LDHs on thermal degradation of IFR/LDHs.

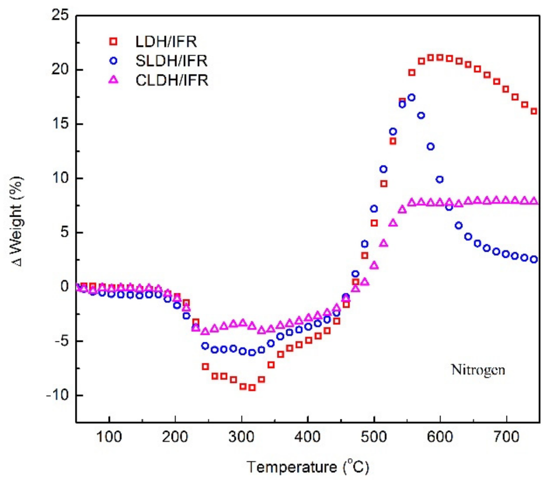


**Figure S2.** ΔWeight versus temperature curves of IFR/LDHs.

CONE testing was performed on PE/IFR/LDHs composites with three kinds of LDHs. The CONE results were shown in Table S4.

**Table S4.** Data of CONE.

| Sample | PE/IFR | PE/IFR/LDH | PE/IFR/SLDH | PE/IFR/CLDH |
|---|---|---|---|---|
| TTI (s) | 44 | 51 | 43 | 45 |
| Flame out (s) | 325 | 511 | 335 | 553 |
| avHRR (kW/m$^2$) | 269 | 151 | 231 | 147 |
| pHRR (kW/m$^2$) | 494 | 374 | 418 | 450 |
| Time to pHRR (s) | 264 | 426 | 274 | 462 |
| THR (MJ/m$^2$) | 90 | 80 | 87 | 86 |
| TSR (m$^2$/m$^2$) | 1840 | 1507 | 1475 | 996 |

The morphological structure of the char after CONE testing was photographed by digital camera in Figure S3

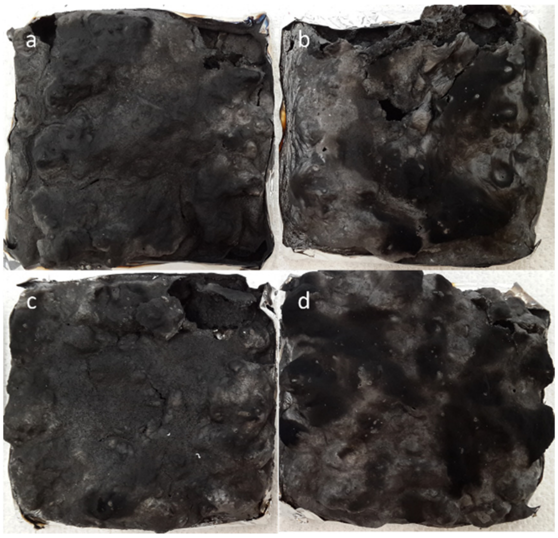


**Figure S3.** The digital photographs of the char after CONE Testing of the composites. (**a**) PE/IFR, (**b**) PE/IFR/LDH, (**c**) PE/IFR/SLDH and (**d**) PE/IFR/CLDH.

The char was investigated by XPS and the results were shown in Table S5.

**Table S5.** Data of XPS.

| Sample | PE/IFR | PE/IFR/LDH | PE/IFR/SLDH | PE/IFR/CLDH |
|---|---|---|---|---|
| C (at.%) | 29.68 | 23.54 | 32.32 | 27.91 |
| N (at.%) | 2.68 | 1.58 | 1.74 | 1.97 |
| O (at.%) | 54.78 | 57.97 | 51.82 | 54.83 |
| Mg (at.%) | 0 | 2.12 | 1.41 | 1.59 |
| Al (at.%) | 0 | 0.83 | 0.79 | 0.72 |
| P (at.%) | 12.86 | 13.96 | 11.92 | 12.98 |
| P:C | 0.43 | 0.59 | 0.37 | 0.47 |
| Mg:Al | - | 2.55 | 1.78 | 2.21 |
| P:Mg | - | 6.58 | 8.45 | 8.16 |
| Mg:C | - | 0.09 | 0.044 | 0.057 |
